# Prevalence of hypertension and its risk factors among cotton textile workers in low- and middle-income countries: a protocol for a systematic review

**DOI:** 10.1186/s13643-020-01364-z

**Published:** 2020-05-02

**Authors:** Naureen Akber Ali, Anam Shahil Feroz

**Affiliations:** 1 School of Nursing and Midwifery, The Aga Khan University, Stadium Road, PO Box 3500, Karachi, 74800 Pakistan; 2 Department of Community Health Sciences, The Aga Khan University, Stadium Road, PO Box 3500, Karachi, 74800 Pakistan

**Keywords:** Blood pressure, Hypertension, Risk factors, Prevalence, Adult workers, Low- and middle- income countries, Systematic review, Cotton textile workers

## Abstract

**Background:**

Cotton workers are exposed to various hazards in the textile industry that might result in different ailments including hypertension (HTN). However, few attempts have been made to systematically review the prevalence of hypertension and its risk factor among cotton textile workers in low-and middle-income countries (LMICs). The objective of this study will be to evaluate the prevalence of hypertension and its risk factors among adult cotton textile workers in low- and middle-income countries.

**Methods:**

We designed and registered a study protocol for a systematic review of descriptive epidemiology data. We will include observational studies (e.g., cross-sectional, cohort, surveys) on the epidemiology of hypertension among adult cotton textile workers in low- and middle-income countries. The primary outcome will be the prevalence of hypertension. Secondary outcomes will be the prevalence of risk factors of hypertension. Literature searches will be conducted in multiple electronic databases (from January 2000 onwards), including PubMed/MEDLINE, CINAHL, Science Direct, and Cochrane Library. Gray literature will be identified through searching conference abstracts, thesis dissertations, and public repositories. Two investigators will independently screen all citations, full-text articles, and abstract data. The study methodological quality (or bias) will be appraised using an appropriate tool. If feasible, we will conduct random effects meta-analysis. Additional analyses will be conducted to explore the potential sources of heterogeneity (e.g., age, gender, years of service, textile department).

**Discussion:**

This systematic review will identify, evaluate, and integrate evidence on the prevalence and risk factors of hypertension among adult cotton textile workers in low- and middle-income countries. Our findings will be made publicly available in a repository and published in a peer-reviewed journal.

**Systematic review registration:**

PROSPERO CRD42020167175

## Background

Globally, hypertension (HTN) is identified as one of the leading causes of mortality and disability [1]. It has led to 7.5 million deaths and accounted for 57 million disability-adjusted life years (DALYs) or 3.7% of total DALYs [2]. Hypertension is characterized as a constant rise of systolic blood pressure and/or diastolic blood pressure of more than or equivalent to 140 mmHg and/or 90 mmHg respectively [3]. Currently, around 1.13 billion individuals are suffering from hypertension globally and two-third of them resides in low- and middle-income countries (LMICs) [4] while LMICs’ selection is based on World Bank’s (WB) 2018 Country Classification list, having GNI per capita between $996 and $3895 [5]. The number of hypertensive people is projected to grow by 70 million individual from 2000 to 2025 in high-income countries (HIC) while it is estimated to grow by > 500 million in LMICs over the same period [6]. A systematic review included data on 1,494,609 adults from 45 countries also showed that there is the highest burden of hypertension (32%) in LMICs and prevalence estimates were found to be higher in elders, overweight/obese, urban settlers, and having informal education [7]. Moreover, worldwide high blood pressure is one of the major risk factors for cardiovascular diseases (CVD) that refers to diseases of the heart and blood vessels [8, 9], stroke, coronary artery disease, and peripheral vascular disease [9]. The burden of CVD across the world is very high and in LMICs, 47% of mortality secondary to CVDs and 44% of CVDs’ burden are attributable to HTN/high blood pressure (BP) [9, 10]. Generally, it is asymptomatic and the primary presentation can be seen with a devastating event like stroke or heart disease [11]. It is estimated that 45% of deaths with heart disease and 51% of deaths with stroke are related to hypertension [12]. This is the reason that it is referred to as the “silent killer” [11].

Worldwide, the textile industry has more than 60 million employees [13] who are involved in the production of fibers, yarns, fabrics, clothing, and textile products for domestic and decoration, along with the technical and industrial purposes [14]. Moreover, the textile industry involves a cluster of related industries that utilize a range of natural fibers (cotton, wool, silk, etc.) and/or synthetic fibers to generate fabric [15] which eventually produces varying amounts of dust [16]. Workers are exposed to various hazards in textile industry that might result in different ailments [17, 18]. Studies reveal high level of oxidative stress in cotton textile workers [19] leading to the development of essential hypertension [20, 21]. A study conducted in Kannur district in India found out that the prevalence of hypertension was 22.3% among cotton textile workers which was equivalent to the prevalence of general population [22]. Furthermore, increasing age, alcohol consumption, family history of hypertension, BMI > 25 kg/m^2^, and high waist-hip ratio were identified as a strong factor of hypertension among cotton workers [22]. Similarly, a study conducted in Dhaka, Bangladesh, notified that both general and central obesity were found to be significantly associated with hypertension [23]. Additionally, factors such as work-related stress, noise pollution, lack of earplug usage, tobacco smoking, and long duration of employment contribute towards the development of hypertension among the cotton textile workers [22, 24–27]. While stress in job also has a negative impact on worker’s cardiovascular health which in turn will affect their job performance [28]. However, the association between cotton dust and increased blood pressure may not be strong, as confounding factors and bias impact could not be excluded [18, 29].

Chronic diseases make workers less productive, more prone to injury that leads to significant suffering, and add huge financial and service challenges on health care systems [23, 30–32]. Furthermore, it is witnessed that collective monetary losses related with the hypertension and diabetes mellitus (DM) in low- and middle-income countries are estimated to be over $7 trillion during the years 2011–2025 that move millions of individuals below the poverty line [33]. Hypertension accounts for the global burden of cardiovascular disease which evidently has huge impact on individuals, organizations, and country in terms of economy [11]. Therefore, early detection of hypertension by identifying its risk factors is essential among high-risk population particularly in LMIC. Thus, the objective of this study will be to evaluate the prevalence of hypertension and its risk factors among adult cotton textile workers in low- and middle-income countries.

## Methods

The present protocol has been registered within the PROSPERO database (registration ID: CRD42020167175). The present study protocol is being reported in accordance with the reporting guidance provided in the Preferred Reporting Items for Systematic Reviews and Meta-Analyses Protocols (PRISMA-P) statement [34] (see PRISMA-P checklist in Additional file 1).

### Eligibility criteria

Studies will be selected according to the following criteria: participants, outcome(s) of interest, study design, and context.

#### Participants (population)

We will include studies involving adult cotton textile workers (regardless of gender). We will exclude adults working in other industries (e.g., oil mill, steel mill, and coal mill).

#### Condition or outcome(s) of interest

The primary outcome will be the prevalence of hypertension (or high blood pressure) indicating the number of people that has the condition divided by the population number at a given point in time. This is often presented as a (prevalence) proportion. We will use author-reported definitions (according to definitions used in the included studies e.g., accepted diagnostic criteria or self-report). Secondary outcomes will be the individual risk factor of hypertension. Potential individual risk factors may include the following: being overweight or obese, physically inactive, using tobacco, excessive alcohol intake, excessive salt (sodium) in the diet, too little potassium in the diet, stress, or certain chronic conditions (such as kidney disease, diabetes, and sleep apnea).

#### Study design and context

Eligible studies will be observational studies (cohort, cross-sectional, or health surveys) reporting prevalence data using validated or non-validated tools and conducted in low- and middle-income countries. Cross-sectional studies will be the most appropriate study design to determine the prevalence of hypertension. Cross-sectional health surveys are typically used to estimate the point prevalence of common conditions of long duration. For cohort studies, only the first phase (cross-sectional) data will be considered. We will exclude randomized trials, quasi-experimental studies, case studies, case series, qualitative studies, systematic review, protocols, commentaries, and editorials.

#### Context/settings

We will include studies conducted in low- and middle-income countries. No limitations will be imposed on publication status (unpublished studies will be eligible for inclusion e.g., conference proceedings, abstracts). We will only include studies published in English, from January 2000 onwards.

### Information sources and search strategy

The primary source of literature will be a structured search of major electronic databases (from January 2000 onwards): MEDLINE (PubMed), CINAHL Plus, Science Direct, and Cochrane Library. The secondary source of potentially relevant material will be a search of the gray or difficult to locate literature, including conference abstracts from selected national or international meeting, thesis dissertations, or documents in public repositories. We will perform hand-searching of the reference lists of included studies, relevant reviews, or other relevant documents. The literature searches will be designed and conducted by the review team. The search terms will be grouped into the following categories of interest: population (e.g., cotton textile workers, factory workers, and cotton workers), epidemiological studies (e.g., prevalence, cross-sectional), outcomes (e.g., hypertension, blood pressure, systolic blood pressure, diastolic blood pressure, high blood pressure and HTN, risk factors, factors, predictors), and settings (e.g., low- and middle-income countries). Additionally, indexed keywords in the Medical Subject Headings (MeSH) will be used in order to ensure uniform search terms. The search strategy will be piloted to ensure sufficient specificity and sensitivity. A draft search strategy for PubMed/MEDLINE is provided in Additional file 2.

### Screening and selection of studies

All articles identified from the literature search will be uploaded into EndNote [35]. Records will be screened by the two reviewers (NA and AF) independently. A pre-defined screening tool will be designed, and a pilot testing will be conducted. First, titles and abstracts of articles returned from initial searches will be screened based on the eligibility criteria outlined above. Second, full texts will be examined in detail and screened for eligibility. Third, references of all considered articles will be hand-searched to identify any relevant report missed in the search strategy. Any disagreements will be resolved by discussion to meet a consensus, if necessary. We will contact the primary author of the study through email in case of any incomplete or missing information. A flow chart showing details of studies included and excluded at each stage of the study selection process will be provided

### Data collection process

Data will be extracted onto a customized sheet in Excel that will be pilot tested prior to initiating the data extraction process. This sheet will be completed by the two independent reviewers (NA, AF) for the eligible studies. Data extraction tables filled by two the reviewers will be compared to confirm that all main findings are included. During the data extraction process, a third assessor (SF) will be involved if any discordant data is witnessed. A pilot data extraction table is provided in Additional file 3. Besides, existing studies on this research domain have been revised to assess items in the extraction form. The form included the primary author, year of publication, study setting, country of study, objective, study design, study population, male proportion, average age, BP measurement method, BP measurement apparatus, prevalence of hypertension, average BP, risk factors of hypertension, co-morbidities, study limitations, included/excluded, and reason for exclusion, quality appraisal of included studies, and reviewer name.

### Evaluation of study quality

The quality of the included studies will be evaluated by standardized quality assessment tool which will be conducted by the two authors (NA, AF). Mixed Methods Appraisal Tool (MMAT) will be used to evaluate the methodological quality of all non-randomized studies [36]. This tool is used to assess several aspects like selection of study participant, study tool, exposure period, missing data, measurement in outcomes, and selection of the reported result. Two reviewers (NA, AF) will rate each study as critical, serious, moderate, or low risk of bias via judgment of the gathered information. If there is limited information then the risk of bias will be categorized as “no information” or the reviewer will contact corresponding study authors for complete information.

### Synthesis of included studies

The data from each paper (e.g., study characteristics, context, participants, outcomes, and findings) will be used to build evidence tables of an overall description of included studies. Crude prevalence estimates (number of cases/sample size) will be presented along with 95% confidence intervals. If feasible and appropriate, prevalence data from primary observational studies will be used to perform random effects meta-analyses. Since heterogeneity is expected a priori, we will estimate the pooled prevalence using the random effects model. The random effects model assumes the study prevalence estimates follow a normal distribution, considering both within-study and between-study variation. Forest plots will be used to visualize the extent of heterogeneity among studies. We will quantify statistical heterogeneity by estimating the variance between studies using *I*^2^ statistic. The *I*^2^ is the proportion of variation in prevalence estimates that is due to genuine variation in prevalence rather than sampling (random) error. *I*^2^ ranges between 0 and 100% (with values of 0–25% and 75–100% taken to indicate low and considerable heterogeneity, respectively). We will also report Cochran *Q* test with a *P* value of < 0.05 considered statistically significant. If sufficient studies are identified and data points are available, potential sources of heterogeneity will be investigated further by subgroup analyses according to baseline characteristics and methodological covariates. We plan to conduct analyses by gender (male vs. female), age (e.g., young adult, middle-age adult, vs. older adult), years of service, and textile department.

### Meta-biases

If data permits, small study effects (publication bias) will be assessed by inspection of the funnel plots for asymmetry and with Egger’s test and Begg’s test, with the results considered to indicate potential small study effects when *P* values < 0.10.

## Discussion

This review will highlight the proportion of hypertension along with its risk factor among cotton textile workers in LMICs. Given that, some of the modifiable factors may be prevented in this high-risk population, we will call on health experts including policy makers, stakeholders, cardiovascular epidemiologist, and occupational health and safety specialists to prioritize policies and commission and conduct screening and awareness programs to improve worker’s health. Besides, this review will highlight an urgent need for further research on hypertension in cotton textile workers that fill in some of the data gaps such as the trends and determinants of hypertension. The review findings will be made publicly available. The results of the review will be disseminated through peer-reviewed publications and presenting at professional conferences either in the form of oral or poster presentations. Any amendments made to this protocol when conducting the study will be outlined and reported in the final manuscript.

## Supplementary information

**Additional file 1:.** PRISMA-P (Preferred Reporting Items for Systematic review and Meta-Analysis Protocols) 2015 checklist: recommended items to address in a systematic review protocol*

**Additional file 2:.** Search strategy for PubMed/MEDLINE

**Additional file 3:.** Data Extraction Table

## Data Availability

Not applicable

## Abbreviations

HTN: Hypertension
DALYs: Disability-adjusted life years
CVD: Cardiovascular disease
LMICs: Lower-middle-income countries
DM: Diabetes mellitus
PRISMA: Preferred Reporting Items for Systematic Reviews and Meta-analyses
