# Supplementary material for: Prevalence of hypertension and its risk factors among cotton textile workers in low- and middle-income countries: a protocol for a systematic review
Source: Syst Rev. 2020 May 2;9:99. doi: 10.1186/s13643-020-01364-z (PMC7196224; doi:10.1186/s13643-020-01364-z)
Supplement: Supplementary file 2 — Additional file 2:. Search strategy for PubMed/MEDLINE [file 13643_2020_1364_MOESM2_ESM.docx]

Additional file 2: Search strategy for PubMed/MEDLINE

| Population | (‘Textile* [Mesh] OR ‘workers*’ OR ‘factory worker*’ OR ‘mill worker’* OR ‘cotton mill worker*’OR ‘ cotton worker*’ [Mesh]) AND |
| --- | --- |
| Outcome | Outcome (‘Hypertension’ OR ‘blood pressure’ OR ‘systolic blood pressure’ OR ‘diastolic blood pressure’ OR hypertension* OR high blood pressure OR HTN OR ‘risk-factors’ OR ‘factors’ OR ‘predictors’) AND |
| Epidemiology Studies | (‘Cross-sectional’ OR ‘cohort’ OR ‘health surveys’ OR ‘surveys’) AND |
| Setting | Studies conducted in low- and middle-income countries (LMICs) |
| Filters | Publication date from 1 January, 2000 to 31 December, 2019; Humans |
