# Supplementary material for: Prevalence of hypertension and its risk factors among cotton textile workers in low- and middle-income countries: a protocol for a systematic review
Source: Syst Rev. 2020 May 2;9:99. doi: 10.1186/s13643-020-01364-z (PMC7196224; doi:10.1186/s13643-020-01364-z)
Supplement: Supplementary file 3 — Additional file 3:. Data Extraction Table [file 13643_2020_1364_MOESM3_ESM.docx]

**Additional file 3: Data Extraction Table**

| **S.No** | **Characteristics** | **Study 1** | **Study 2** | **Study 3** | **Study 4** | **Study 5** | **Study 6** | **Study 7** | **Study 8** |
| --- | --- | --- | --- | --- | --- | --- | --- | --- | --- |
|  | Study Author & Year |  |  |  |  |  |  |  |  |
|  | Setting & Country |  |  |  |  |  |  |  |  |
|  | Study Aim |  |  |  |  |  |  |  |  |
|  | Study Design |  |  |  |  |  |  |  |  |
|  | Study Population |  |  |  |  |  |  |  |  |
|  | Male proportion |  |  |  |  |  |  |  |  |
|  | Average age |  |  |  |  |  |  |  |  |
|  | Average BP |  |  |  |  |  |  |  |  |
|  | Bp Measurement methods |  |  |  |  |  |  |  |  |
|  | Bp Measurement apparatus |  |  |  |  |  |  |  |  |
|  | Prevalence |  |  |  |  |  |  |  |  |
|  | Risk-Factors |  |  |  |  |  |  |  |  |
|  | Co-Morbidities |  |  |  |  |  |  |  |  |
